# The Relationship between Risk Levels of Breast Cancer and Use of Early Diagnosis and Screening Services in Healthcare Workers in Turkey

**DOI:** 10.18502/ijph.v49i7.3582

**Published:** 2020-07

**Authors:** Ayla ACIKGOZ, Selda YORUK, Hulya TURKMEN, Gul ERGOR

**Affiliations:** 1. Vocational School of Health Services, Dokuz Eylül University, Izmir, Turkey; 2. Department of Midwifery, Balikesir School of Health, Balıkesir University, Balikesir, Turkey; 3. Department of Public Health, School of Medicine, Dokuz Eylul University, Izmir, Turkey

**Keywords:** Healthcare workers, Breast cancer, Mammography, Breast cancer screening, Breast self-examination

## Abstract

**Background:**

This study aimed to determine the factors affecting early diagnosis and screening behaviors of healthcare workers concerning breast cancer and the breast cancer risk levels using the risk identification model and to evaluate the relationship between breast cancer risk levels and early diagnosis and screening behaviors.

**Methods:**

Overall, 466 healthcare workers from Balikesir Province, Turkey participated in this cross-sectional study. Data were collected thanks to a questionnaire prepared by the researchers. Cuzick-Tyrer model was utilized to determine breast cancer risk levels.

**Results:**

78.1% of the healthcare workers regularly perform breast self-examination (BSE), 11.6% had clinical breast examination (CBE), 7.7% had breast ultrasound scan and 4.5% had mammography. BSE behavior increased, as education level got higher. Mammography screening behavior increased in those aged 40 yr and older and those with breast or ovarian cancer history in their family. There was not any relationship between breast cancer risk levels and early diagnosis and screening behaviors.

**Conclusion:**

Early diagnosis and screening behaviors of healthcare workers were low concerning breast cancer. Age, education level and family history are the most prominent factors affecting early diagnosis and screening behaviors of healthcare workers. Informing healthcare workers on breast cancer risk factors and screening can make positive contributions to them and the public through them.

## Introduction

Breast cancer is the most frequent type of cancer and the leading cause of mortality in women in Turkey as it is in the world (1, 2). Breast cancer incidence is lower in women under the age of 40, however it increases with age (3). Gender, that is to say being female, and age are to two main factors that increase breast cancer risk. The incidence of age-specific breast cancer increases rapidly starting from the age of 40 (1, 2). Breast cancer incidence and mortality varies from one country to another. The fact that developed countries have better early diagnosis and treatment possibilities thanks to screening mammography is one of the main reasons constituting these important differences among countries (4, 5). Early diagnosis is the most important factor in the course of disease.

WHO suggests the implementation of community-based breast cancer screening programs in line with the countries' possibilities (4). Breast Self-Examination (BSE), Clinical Breast Examination (CBE), Breast Ultrasound (US) and mammography are the primary methods proposed for the early diagnosis of breast cancer. However, there are on-going discussions regarding early diagnosis and screening age and frequency regarding breast cancer. All asymptomatic women perform BSE regularly every month between the ages of 20–39 and have CBE every one to three years, that women in the age group of 40 and over have mammography once in 1 to 2 years and that women have a breast US to confirm the mammography in intense breast cases and case of breast cancer suspicion before the age of 40 (3, 6, 7).

Determining the risk of breast cancer in women is very important in terms of intervention and prevention to reduce the risk of breast cancer (8). There are different models for identification of breast cancer risk levels (3, 8, 9). In these models, breast cancer risk is mathematically calculated based on the known breast cancer risk factors (9, 10). Using the Cuzick-Tyrer model, 10-year and lifetime breast cancer risk can be calculated by taking into account variables related to family history, hormonal factors, benign breast disease, BRCA mutation. While forming the Cuzick-Tyrer model, some risk factors, which are not included in other models, such as body mass index, age of menopause, duration of hormone replacement therapy (HRT), presence of in situ carcinoma, presence of breast and ovarian cancer in second or third-degree relatives, age of diagnosis and presence of breast cancer in male relatives have been taken into consideration. Therefore, this model is considered to be the most sensitive and best model that is constantly renewed for breast cancer prediction (8–10).

Lifestyles of healthcare workers regarding the risk of breast cancer and their use of early diagnosis methods are indicators of their awareness on this matter. Because midwives and nurses are at higher risk of cancer due to working conditions (11–13). In addition, healthcare workers are a direct source of medical information for the public and patients (8). Nurses and midwives working at every stage of the healthcare system make direct contact with the public. Midwives and nurses provide information and support regarding medical problems including cancer (6, 8). The positive attitudes and behaviors of healthcare workers regarding the early diagnosis and screening of breast cancer may contribute both to themselves and the people in their service area regarding cancer prevention (8, 14).

This study aimed to determine the factors affecting early diagnosis and screening behaviors of healthcare workers concerning breast cancer and the breast cancer risk levels using the risk identification model and to evaluate the relationship between breast cancer risk levels and early diagnosis and screening behaviors.

## Materials and Methods

### Participants

The population of this cross-sectional study consists of female midwives and nurses (N= 680) working in two different hospitals providing healthcare services in Balikesir Province, Turkey. These hospitals are the largest public hospitals that have the largest number of nurses and midwives in the province. It was aimed to reach to the entire population by not doing sample selection. Questionnaires were collected in sealed envelopes from the participant at the institutions where they work. Twelve healthcare workers were excluded from the research because they did not complete the questionnaire fully. In total, 466 healthcare workers completed the questionnaire fully.

### Measurements and Definitions

Data were collected thanks to a questionnaire prepared by the researchers. The questionnaire included questions on participants' sociodemographic characteristics, lifestyle, early diagnosis and screening behaviors and breast cancer risk levels. Cuzick-Tyrer model was utilized to determine breast cancer risk levels (10). According to this model, those who have higher risk than the others at the same age group are classified as "high risk" and those at same or lower risk are classified as "low risk". The questions asked in the risk calculation program were added to the questionnaire.

Institutional approval was obtained from the Local Ethics Committee. The study was conducted in accordance with the Declaration of Helsinki. Informed consent was obtained from the participants in the study. The data collection stage of the research took place from Jan to Sep 2017.

### Statistical analysis

The data were analyzed through the SPSS for Windows 20.0 (Chicago, IL, USA). Continuous variables were presented with their averages and standard deviations, categorical variables were presented with their number and percentage distributions. Odds Ratio (OR) and 95% confidence interval (CI) were calculated to assess the relationship between independent variables (age, education, marital status, history of chronic disease, cancer history in family, breast cancer risk) and dependent variables (BSE, CBE, US, mammography). Pearson chi-square test, chi-square for trend test, Fisher's exact test and logistic regression analysis were used for statistical analysis. In an attempt to examine the factors affecting BSE behavior, a logistic regression model, which includes age, education level, presence of chronic diseases and family history of breast or ovarian cancer, was established. In an attempt to analyze the factors affecting mammography behavior, a logistic regression model, which includes age, education level and family history of breast or ovarian cancer, was established. Statistical significance level was considered to be *P*<0.05.

## Results

Overall, 466 healthcare workers participated in this study. The average age of the participants was 33.3±7.4 (Min=22, Max=55). Out of all participants, 78.1% stated to have regular BSE, 11.6% to have CBE, 7.7% to have breast US and 4.5% to have mammography. Approximate breast cancer risks of participants were estimated based on the Cuzick-Tyrer model. 92.1% of the participants had low 10-year breast cancer risk, whereas 7.9% had high risk. Lifetime breast cancer risk was low for 91.0% of the participants and high for 9.0%.

A significant relationship was identified between participants' age, education level, history of chronic illness and BSE behavior. As age advances, BSE behavior decreases significantly (*P*<0.05). BSE behavior is reduced in the age group of ≥40 yr (Crude OR: 0.50 95% CI: 0.27–0.92). As the level of education increases, BSE behavior increases (*P*<0.05). Those who have a chronic illness tend to have BSE more often (*P*<0.05) (Table 1). In the logistic regression model designed to examine the factors affecting the BSE behavior, participants' education level and presence of chronic illnesses were found to be the most significant factors. BSE behavior was found to be seen 1.87 times (Adjusted OR:1.87, 95%CI=1.03–3.42) more in women with a bachelor degree than high school graduates and it has decreased by 68% in patients with chronic illnesses (Adjusted OR: 0.32, 95%CI= 0.17–0.62) (not shown in the tables). As healthcare workers age, they start having more CBEs (*P*<0.05) (Table 2). Except for their age, there was no relationship between other characteristics of healthcare workers and their behavior of having CBE. The age group of ≥40 yr has breast US 9.41 times more than the age group of 20–29 yr (*P*<0.05) (Table 3). Except for their age, there was no relationship between other characteristics of healthcare workers and their behavior of having breast US.

**Table 1:** The relationship between certain characteristics of the participants, calculated breast cancer risk and BSE behavior

***Factors***		***BSE***		***P***	***Crude OR (%95 CI)***	***P***
		***Yes***	***No***			
		***(n=364)***	***(n=102)***			
		n (%)	n (%)			
Age(yr)	20–29	136 (81.0)	32 (19.0)	0.043^‡^	1.00	
	30–39	172 (79.6)	44 (20.4)		0.92 (0.55–1.52)	0.375
	≥40	56 (68.3)	26 (31.7)		0.50 (0.27–0.92)	0.029
Education	High school	51 (67.1)	25 (32.9)	0.031^‡^	1.00	
	College	129 (79.6)	33 (20.4)		1.91 (1.02–3.53)	0.040
	University	184 (80.7)	44 (19.3)		2.04 (1.13–3.65)	0.017
Marital status	Married	255 (78.9)	68 (21.1)	0.512^*^	1.00	
	Others	109 (76.2)	34 (23.8)		0.85 (0.53–1.36)	0.512
History of chronic disease	No	338 (80.7)	81 (19.3)	0.001^*^	1.00	
	Yes	26 (55.3)	21 (44.7)		0.29 (0.15–0.56)	0.001
Breast/ovarian cancer history in family	No	331 (78.1)	93 (21.9)	0.940^*^	1.00	
	Yes	33 (78.6)	9 (21.4)		1.03 (0.48–2.35)	0.963
Cuzick-Tyrer model (ten-year breast cancer risk)^**^	Low	333 (77.6)	96 (22.4)	0.384^*^	1.00	
	High	31 (83.8)	6 (16.2)		1.48 (0.62–4.02)	0.399
Cuzick-Tyrer model (lifetime breast cancer risk)^**^	Low	327 (77.1)	97 (22.9)	0.101^*^	1.00	
	High	37 (88.1)	5 (11.9)		2.19 (0.88–6.44)	0.094

‡ Chi-square for trend,

* Pearson Chi-square

** Camparison of individual risk with respect to general population

**Table 2:** The relationship between certain characteristics of the participants, calculated breast cancer risk and CBE behavior

***Factors***		***CBE***		***P***	***Crude OR (%95 CI)***	***P***
		***Yes***	***No***			
		***(n=54)***	***(n=412)***			
		**n (%)**	**n (%)**			
Age(yr)	20–29	8 (4.8)	160 (95.2)	0.001^‡^	1.00	
	30–39	13 (6.0)	203 (94.0)		1.28 (0.51–3.32)	0.605
	≥40	33 (40.2)	49 (59.8)		13.47(5.83–31.08)	0.001
Education	High school	12 (15.8)	64 (84.2)	0.404^*^	1.00	
	College	19 (11.7)	143 (88.3)		0.70 (0.32–1.59)	0.196
	University	23 (10.1)	205 (89.9)		0.59 (0.28–1.31)	0.095
Marital status	Married	42 (13.0)	281 (87.0)	0.151^*^	1.00	
	Others	12 (8.4)	131 (91.6)		0.61 (0.30–1.18)	0.075
History of chronic disease	No	50 (11.9)	369 (88.1)	0.487^*^	1.0	
	Yes	4 (8.5)	43 (91.5)		0.68 (0.20–1.85)	0.258
Breast/ovarian cancer history in family	No	47 (11.1)	377 (88.9)	0.281^*^	1.00	
	Yes	7 (16.7)	35 (83.3)		1.60 (0.62–3.69)	0.147
Cuzick-Tyrer model (ten-year breast cancer risk)^**^	Low	50(11.7)	379 (88.3)	0.878^*^	1.00	
	High	4 (10.8)	33 (89.2)		0.91 (0.26–2.52)	0.461
Cuzick-Tyrer model (lifetime breast cancer risk)^**^	Low	50 (11.8)	374 (88.2)	0.661^*^	1.00	
	High	4 (9.5)	38 (90.5)		0.78 (0.23–2.14)	0.351

‡ Chi-square for trend,

* Pearson Chi-square

** Camparison of individual risk with respect to general population

**Table 3:** The relationship between certain characteristics of the participants, calculated breast cancer risk and breast US behavior

***Factors***		***Breast US***		***P***	***Crude OR (%95 CI)***	***P***
		***Yes***	***No***			
		***(n=36)***	***(n=430)***			
		**n (%)**	**n (%)**			
Age(yr)	20–29	7 (4.2)	161 (95.8)	0.001^*^	1.00	
	30–39	5 (2.3)	211 (97.7)		0.54 (0.15–1.79)	0.321
	≥40	24 (29.3)	58 (70.7)		9.41 (3.95–24.68)	0.001
Education	High school	8 (10.5)	68 (89.5)	0.400^*^	1.00	
	College	14 (8.6)	148 (91.4)		0.80 (0.32–2.11)	0.637
	University	14 (6.1)	214 (93.9)		0.55 (0.22–1.45)	0.220
Marital status	Married	27 (8.4)	296 (91.6)	0.441^*^	1.00	
	Others	9 (6.3)	134 (93.7)		0.73 (0.32–1.57)	0.454
History of chronic disease	No	33 (7.9)	386 (92.1)	0.716^*^	1.00	
	Yes	3 (6.4)	44 (93.6)		0.79 (0.18–2.46)	0.767
Breast/ovarian cancer history in family	No	32 (7.5)	392 (92.5)	0.647^*^	1.00	
	Yes	4 (9.5)	38 (90.5)		1.28 (0.37–3.60)	0.625
Cuzick-Tyrer model (ten-year breast cancer risk)^**^	Low	32 (7.5)	397 (92.5)	0.464^*^	1.00	
	High	4 (10.8)	33 (89.2)		1.50(0.43–4.23)	0.463
Cuzick-Tyrer model (lifetime breast cancer risk)^**^	Low	32 (7.8)	392 (92.5)	0.647^*^	1.00	
	High	4 (9.5)	38 (90.5)		1.28 (0.37–3.60)	0.625

* Pearson Chi-square

** Camparison of individual risk with respect to general population

As the age advances, behavior of having mammography increases as well (*P*<0.05). Mammography behavior is 3.43 times more in those who have family history of breast or ovarian cancer (*P*<0.05) (Table 4). In the logistic regression model designed to examine the factors affecting the mammography behavior, participants' age was found to be the most significant factor.

**Table 4:** The relationship between certain characteristics of the participants, calculated breast cancer risk and mammography behavior

***Factors***		***Mammography***		***P***	***Crude OR (%95 CI)***	***P***
		***Yes***	***No***			
		***(n=21)***	***(n=445)***			
		**n (%)**	**n (%)**			
Age(yr)	20–29	1 (0.6)	167 (99.4)	0.001‡	1.00	
	30–39	7 (3.2)	209 (96.8)		5.57 (0.85–12.70)	0.079
	≥40	13 (15.9)	69 (84.1)		31.07 (5.28–67.85)	0.001
Education	High school	5 (6.6)	71 (93.4)	0.626^*^	1.00	
	College	7 (4.3)	155 (95.7)		0.62 (0.19–2.28)	0.470
	University	9 (3.9)	219 (96.1)		0.58 (0.18–1.98)	0.361
Marital status	Married	16 (5.0)	307 (95.0)	0.484 ^*^	1.00	
	Others	5 (3.5)	138 (96.5)		0.69 (0.22–1.87)	0.507
History of chronic disease	No	20 (4.8)	399 (95.2)	0.407 ^*^	1.00	
	Yes	1 (2.1)	46 (97.9)		0.43 (0.02–2.44)	0.455
Breast/ovarian cancer history in family	No	16 (3.8)	408 (96.2)	0.032 ^†^	1.00	
	Yes	5 (11.9)	37 (88.1)		3.43 (1.07–9.61)	0.039
Cuzick-Tyrer model (ten-year breast cancer risk) ^**^	Low	18 (4.2)	411 (95.8)	0.271^*^	1.00	
	High	3 (8.1)	34 (91.9)		2.01 (0.45–6.65)	0.303
Cuzick-Tyrer model (lifetime breast cancer risk) ^**^	Low	18 (4.2)	406 (95.8)	0.388^*^	1.00	
	High	3 (7.1)	39 (92.9)		1.73 (0.39–5.69)	0.401

‡ Chi-square for trend,

† Fishers’ exact test,

* Pearson Chi-square

** Camparison of individual risk with respect to general population

While mammography behavior remains the same in the age groups of 20–29 yr and 30–39 yr, the age group of ≥40 yr is found to have mammography 28.66 times (Adjusted OR:28.66, 95%CI= 3.63–226.24) more often (not shown in the tables). Except for age and family history of breast or ovarian cancer, there was no relationship between other characteristics of healthcare workers and their behavior of having mammography.

## Discussion

In matters of prevention from breast cancer and early diagnosis, the public needs to be informed about these notions. Healthcare workers play the principal role in raising the awareness of the public. Attitudes and behaviors of healthcare workers, who set an example for the public in respect to prevention from breast cancer and early diagnosis, towards cancer screening programs affect the success of national cancer screening programs (2, 8, 14). In this study, BSE, CBE, US and mammography behaviors of female healthcare workers working in hospitals and their breast cancer risk levels were identified. The relationship between estimated breast cancer risk levels and early diagnosis and screening behaviors was analyzed.

BSE is an easy, harmless, no-cost early diagnosis method that every woman can perform. Every woman should perform BSE regularly to be aware of any abnormal changes in their breasts (4). 78.1% of the participants stated that they performed BSE regularly. This rate is similar to the results of a study conducted on the general population in a city center in Turkey (15) and is higher than the results of a study conducted in China (16). Our finding is lower than that of a study conducted on healthcare workers in Turkey (17) and higher than that of studies conducted on healthcare workers in Singapore, Brazil, Taiwan and Turkey (18–24).

The healthcare workers participating in this study are young, have higher education level and have history of chronic illnesses are related to their behaviors of having BSE. In studies conducted on healthcare workers, those, who were young and had higher education level, performed BSE more often, which conforms to our findings (17–20). In contrast with our results, those; married (17), had breast-related diseases or family history of breast cancer (18), were well-informed about the breast cancer (24), were more experienced in nursing and provided care for patients with breast cancer (19), tended to perform BSE more often. Having a higher education level might have increased BSE awareness of healthcare workers. The fact that younger women perform BSE more often evokes the idea that they are more aware regarding BSE, since mammography and screening start after the age of 40 years.

CBE is the palpation of axilla and all breast tissues by a physician or other trained healthcare personnel and the assessment of breast cancer findings. Especially women, who are under 40 yr of age, should have CBE regularly (4, 6). 11.6% of the healthcare workers participating in this study have had CBE. In other countries, CBE rate in healthcare workers is 16.1% to 88.0% (17, 18, 21, 24–26). As for the general public in Turkey, a study revealed that CBE rate was found to be 33.0% (22). The fact that majority of the participants of these studies were over 40 yr of age may have affected the rates. CBE is usually carried out as a routine procedure prior to mammography screening by doctors in Turkey. In this study, as healthcare workers aged, their CBE behaviors increased significantly. Young people regularly perform BSE and the absence of any sign of breast cancer may be the causes of low rates of CBE behavior. In the literature, women under the age of 40 (26) and women with low breast cancer risk perception (15) were observed to have less CBE. The breast cancer screening is influenced by factors such as age, education level, socioeconomic characteristics and the presence of an effective screening program in the country (4, 14). Except for their age, there was no relationship between other characteristics of healthcare workers and their behavior of having CBE. Healthcare workers are individuals who should perform CBE. In Brazil, the majority of nurses and physicians suggested their patients to have CBE, however that they performed CBE less often as individuals (27).

7.7% of the healthcare workers participating in our study have had breast US. In other countries, breast US rate in healthcare workers is 33.0% to 92.0% (21, 23, 25). As for the general public in China, breast US rate was found to be 33.7% (16). Analyzing the studies in which breast US rates are high, it was seen that the average age of the participants was high compared to our participants. This is also supported by the fact that women in the age group of ≥40 yr participating in our study have breast US 9.4 times more often. Healthcare institutions and organizations recommend that women aged 40 yr and older should have mammography once in 1 to 2 years (2–4, 6, 7, 14). In this study, the mammography rate of participants aged 40 yr and older was 15.9%. When all participants are included, this rate is only 4.5%. Mammography rates were found to be higher than one (17) and lower than other studies conducted on healthcare workers in Turkey (18, 23, 24). In other countries, mammography rate in female healthcare workers is 14.9% to 60.0% (19–21, 25, 26, 28). The socioeconomic and cultural structure of the countries affects mammography behaviors in breast cancer screening (5, 14). Furthermore, working areas of healthcare workers may also affect screening behaviors. In the study conducted on primary healthcare workers, mammography rate of participants was lower than our results (17). There are not many cancer patients in the population provided with services might have affected this behavior. On the other hand, the study conducted on primary healthcare workers in Brazil (20) and Saudi Arabia (26) revealed that the rate of having mammography was higher than our result. The majority of healthcare workers participating in these studies are over 40 yr old might have affected the results.

Except for their age and history of breast or ovarian cancer, there was no relationship between other characteristics of healthcare workers and their behavior of having mammography. In the age group of ≥40 yr, mammography screening behavior is observed 28 times more often. This finding is consistent with the literature. Studies conducted in other countries ascertained that as age advanced, mammography behavior of healthcare workers increased (19, 20, 23, 26, 28, 29). Healthcare workers should be informed that mammogram screening should be initiated at 40 yr of age. In Brazil, healthcare workers, who had mammography regularly, suggested their patients to have mammography regularly as well (27).

A study conducted on women living in four different cities in China concluded that women, who worked, were married and had higher education levels, performed more breast screening practices (16). Although there is no relationship found between education levels of healthcare workers and mammography behaviors in our study, there are some studies found a significant relationship between those two factors in the literature (26, 29).

Though there is not a proven genetic mutation, approximately 10%–20% of breast cancer patients have family history (3, 9). This study found out that those who had family history breast or ovarian cancer had mammography screening more often. Only a few studies conducted on healthcare workers addressed mammography behaviors of those with a family history. In these studies, no positive relationship between family history and breast cancer screening behaviors was found (18, 23). Those who have breast cancer histories in their family have a higher risk of breast cancer. Moreover, these women perceive individual breast cancer risk to be high. This perception increases their breast screening behavior (15, 22). However, no relationship between the calculated risk level of breast cancer and mammography behavior was found in this study. Since family history of breast cancer increases the risk of breast cancer, those with a family history should be informed to have regular mammography starting from earlier ages than suggested.

## Conclusion

This study did not ascertain any relationship between breast cancer risk levels and early diagnosis and screening behaviors. Age, education level and family history are the most prominent factors affecting early diagnosis and screening behaviors of healthcare workers. Healthcare workers from all stages of the healthcare industry should be able to lead the public and provide consultation services with respect to breast cancer screening (6, 7). It is recommended to raise the awareness of healthcare workers on breast cancer in line with the national breast cancer screening standards and to make them gain the habit of early diagnosis and screening. Interventional studies aimed at elimination of the breast cancer screening barriers of healthcare workers can be conducted. In women diagnosed with breast cancer, it may be recommended to examine retrospective screening behaviors.

## Ethical considerations

Ethical issues (Including plagiarism, informed consent, misconduct, data fabrication and/or falsification, double publication and/or submission, redundancy, etc.) have been completely observed by the authors.
